# Profiling major volatile components in apricot fruit sheds light on the molecular mechanisms underlying low-temperature-mediated volatile release

**DOI:** 10.1016/j.fochms.2026.100409

**Published:** 2026-05-09

**Authors:** Hua Wang, Pei Sun, Yuan Yang, Wenjian Yu, Yanhui Kang, Maofu Li, Shuting Zhou, Xiangyi Sun, Min Jin, Wanmei Jin, Haoyuan Sun, Yuzhu Wang

**Affiliations:** a Beijing Academy of Agriculture and Forestry Sciences, Beijing 100097, PR China; b Key Laboratory of National Forestry and Grassland Administration on Apricot, China; c Beijing Engineering Research Center of Functional Floriculture, Beijing 100093, PR China

**Keywords:** Apricot, Volatile esters, Aroma loss, Low-temperature storage, Fatty acid metabolism, Lipoxygenase (LOX), Alcohol acyltransferase (AAT)

## Abstract

Storage of ripe apricot (*Prunus armeniaca*) fruit at low temperatures results in the loss of volatile aroma compounds. Here, we analyzed volatile compounds in fruit from the apricot cultivars ‘Chuanzhihong’ and ‘Mituoluo’ during ripening and identified esters as key contributors to fruit aroma. The cultivars accumulated different profiles of volatiles during ripening. Transcriptome analysis identified more than 20,000 genes that were differentially expressed during fruit development and a cluster of genes that were enriched in KEGG pathways associated with fatty acid–derived ester biosynthesis. The key ester biosynthetic genes *LOX* and *AAT* were upregulated during fruit ripening but suppressed at low temperatures. Volatile emissions dropped below 10 °C and partially recovered at 25 °C, but LOX and AAT enzymatic activities failed to recover, leading to diminished aroma. Storage at 0–5 °C caused a strong and largely irreversible suppression of ester accumulation, even after rewarming.

## Highlights

• Esters are the major contributors to apricot fruit aroma during ripening.
• Fatty acid metabolism underlies ripening-dependent volatile ester formation.
• Cold storage markedly reduces ester emissions below 10 °C.
• LOX and AAT activities fail to recover after prolonged low-temperature storage.
• Persistent aroma loss occurs during postharvest cold storage.

## 1 Introduction

Apricot (*Prunus armeniaca* L.), belonging to the Rosaceae family, is the third most widely cultivated stone fruit crop worldwide and an economically important fruit species. Apricot fruit is highly appreciated by consumers for their high nutritional value, which includes abundant vitamins, minerals, and antioxidant compounds, as well as their distinctive aroma (García-Gómez et al., 2021; Ortuño-Hernández et al., 2025). Aroma is a key quality attribute of apricot fruit and is primarily determined by the composition and abundance of volatile organic compounds.

Fruit volatile compounds consist of a set of low molecular-weight, volatile substances with low boiling points. These volatile compounds play roles in repelling pest invasion and contribute to abiotic stress resistance (Clavijo McCormick et al., 2023; Lin et al., 2021). Volatile metabolites in fruit are classified into three major categories: benzene/phenylpropanoid compounds, terpenoids, and fatty acid derivatives (Zhu, Liao, et al., 2022). Benzene/phenylpropanoid compounds are primarily synthesized by the cinnamic acid pathway, which starts with shikimate as a precursor. Phenylalanine ammonia-lyase and phenylacetaldehyde synthase compete for phenylalanine, catalyzing its deamination to form trans-cinnamic acid. Cinnamic acid is then converted to benzoyl co-enzyme A (benzoyl-CoA) through a series of enzymatic reactions such as hydration and oxidation; benzoyl-CoA is converted into phenethyl benzoate via catalysis by benzyl alcohol/phenylethyl alcohol acyltransferase. Finally, benzoic acid methyltransferase catalyzes the formation of methyl benzoate. Methylation, hydroxylation, acetylation, and other modifications of benzene and phenylpropane generate a diverse array of benzene and phenylpropanoid aromatic compounds (Ma et al., 2025).

Isopentenyl diphosphate (IPP) is a common precursor of terpenoids (Yu et al., 2023). IPP is synthesized through the plastid-localized methylerythritol phosphate pathway and the cytosolic mevalonate pathway (Pu et al., 2021). IPP is converted to the direct precursors of terpenoids, namely geranyl diphosphate, farnesyl diphosphate, and geranylgeranyl pyrophosphate, which are subsequently converted into a wide variety of monoterpenes, sesquiterpenes, and diterpenes by monoterpene synthases, sesquiterpene synthases, and diterpene synthases, respectively (Liu et al., 2022). Some terpenoids form structurally diverse derivatives such as terpenols, terpenyl esters, terpenals, terpenones, and terpenoid oxides following their conversion by different types of enzymes (cytochrome P450s, dehydrogenases, reductases, to name a few). In fact, terpenoids are regarded as the largest class of plant secondary metabolites (Ninkuu et al., 2021; Zhang et al., 2023).

Fatty acid derivatives include low molecular weight alcohols, aldehydes, and esters. Their biosynthesis starts with unsaturated linolenic acid and linoleic acid as precursors, forming hydroperoxides via catalysis by lipoxygenase (LOX). The resulting hydroperoxides are further converted into C6 and C9 volatile aldehydes by hydroperoxide lyase (HPL). Subsequently, these aldehydes are converted into the corresponding esters by alcohol dehydrogenase (ADH) and alcohol acetyltransferase (AAT) (Bao et al., 2025).

In the biosynthesis of fatty acid–derived volatile flavor compounds, LOX primarily catalyzes the oxidation of double bonds in polyunsaturated fatty acids to produce hydroperoxides such as linoleic acid hydroperoxide, and linolenic acid hydroperoxide (Lippolis et al., 2023; Ortuño-Hernández et al., 2024). These hydroperoxides serve as key precursors for the biosynthesis of various volatile compounds and can be further decomposed into aldehydes (hexanal, and hexenal) or alcohols (hexenol) under the action of other enzymes (Lippolis et al., 2023; Sánchez-Ortiz et al., 2018). These products confer a mild aroma to foods and may also form ketones, esters, and other volatile compounds (Pan et al., 2025). In summary, LOX provides core precursors for the biosynthesis of volatile compounds by initiating the fatty acid oxidative metabolism pathway and is regarded as a key regulatory enzyme in the formation of natural plant flavors (Zhu, Wen, et al., 2022). The core function of AAT is to catalyze the acetylation reaction between alcohols and acetyl-CoA, producing acetates and CoA. AAT, a key enzyme in ester biosynthesis, is mainly involved in the production of esters such as ethyl acetate, hexyl acetate, butyl acetate, and phenethyl acetate.

The biosynthesis of the lactone γ-decanolide involves the catalytic activities of multiple enzymes. In plants, γ-decanolide biosynthesis begins with fatty acids as initial precursors, which are subsequently converted into specific hydroxy fatty acid intermediates. These hydroxy fatty acids enter the peroxisomes and undergo fatty acid β-oxidation, which involves dehydrogenation, hydration, re-dehydrogenation, and thiolysis reactions, leading to the shortening of the fatty acid carbon chain by two carbon atoms in each cycle to form 4-hydroxydecanoic acid. Under the action of cyclase, hydroxyl and carboxyl groups undergo intramolecular esterification to form a cyclic ester structure, ultimately producing γ-decanolide (Zhang et al., 2024). For example, in peach (*Prunus persica*) fruit, the alcohol acetyltransferase PpAAT1 catalyzes the linkage between the hydroxyl group of 4-hydroxydecanoyl-CoA and an acyl group to generate γ-decanolide (Peng et al., 2020).

The complex formation and accumulation of volatile compounds in apricot fruit are regulated by genetic, environmental, and other factors (Karabulut et al., 2018). Volatile formation in apricot fruit is a complex trait governed by genetic, environmental, and postharvest factors (Karabulut et al., 2018). Although low-temperature storage is widely used to extend shelf life, it frequently impairs aroma development, thereby reducing fruit quality and consumer acceptance. The molecular mechanisms underlying cold-induced changes in aroma remain largely unclear. Emerging evidence indicates that low temperature disrupts volatile biosynthesis by modulating key metabolic pathways. In banana, cold storage suppresses ester production and alters amino acid and fatty acid metabolism via downregulation of genes in volatile-related pathways, particularly lipoxygenase (LOX) (Zhu et al., 2018). Similarly, in peach, low temperature reduces volatile accumulation and affects LOX-associated gene expression, highlighting a conserved regulatory role in aroma formation (Zhang et al., 2011). Despite these advances, studies focusing on apricot are still limited, particularly regarding the integration of volatile metabolism and gene expression under low-temperature conditions. Apricot fruit is consumed fresh or processed into various products such as preserved apricots, dried apricots, apricot juice, and canned apricots. The levels of volatile compounds in apricot fruit increase significantly during development. However, ripe apricots are prone to decay. Therefore, low-temperature storage is commonly used for postharvest preservation, but this method often leads to the loss of volatile flavor compounds. However, few reports describe how low temperatures affect the volatile flavor compounds of stored apricots. Therefore, a deeper understanding of the molecular basis of cold-induced changes in aroma is essential for improving postharvest fruit quality.

In this study, we measured volatile compounds in apricot fruit at different developmental stages and identified esters as key contributors to their aroma. The expression patterns of genes encoding LOX and AAT were consistent with the profile of ester accumulation. Low temperatures significantly inhibited the release of volatile compounds from ripe apricots, with 10 °C being a critical low-temperature threshold. Low temperatures repressed the expression levels of key genes involved in volatile compound biosynthesis and the activity of the corresponding key biosynthetic enzymes. Consequently, low temperatures decrease the release of volatile compounds, leading to the loss of flavor in stored apricots.

## 2 Materials and methods

### 2.1 Plant materials and temperature conditions

The apricot cultivars ‘Mituoluo’ and ‘Chuanzhihong’ were used in this study. Both ‘Chuanzhihong’ and ‘Mituoluo’ are traditional Chinese apricot cultivars widely cultivated in China. ‘Mituoluo’ exhibited a rich volatile profile and ‘Chuanzhihong’ had aldehydes as the dominant volatile compounds (Zhang et al., 2014). The cultivars were grown under natural conditions in an experimental field in Yanqing district, Beijing, China, under natural conditions (116.180597°E, 40.574211°N). The annual mean temperature in Yanqing was 9.9 °C and the warmest month was July with a mean temperature of 24.5 °C. Average annual precipitation was 467 mm, with about 44% falling between June and September (Xu et al., 2019). At maturity, ‘Mituoluo’ exhibited an average single fruit weight of 68.9 g, with a vertical diameter of 4.9 cm, transverse diameter of 5.4 cm, and lateral diameter of 5.3 cm. The ripe fruits are round and yellow, with a slight reddish blush on the sun-exposed side, and are characterized by abundant aromatic volatile compounds. In the Yanqing, the cultivar ripens around July 10th. Also at maturity, ‘Chuanzhihong’ exhibited an average single fruit weight of 52.5 g, with a vertical diameter of 5.0 cm, transverse diameter of 4.7 cm, and lateral diameter of 4.6 cm. Originating from Hebei Province, China, the flesh is orange-yellow, firm and crisp, with fine and sparse fibers, low juice content, and a sweet–sour taste. The ripe fruits are oblong, with a bright red peel at maturity; the ground color is orange-yellow, and approximately three-fourths of the sun-exposed surface develops purplish-red pigmentation. This cultivar ripens around July 20th in the Yanqing.

For sampling, three healthy and uniformly growing trees per cultivar were selected as biological replicates. Fruits were collected at the mature stage during the harvest season (July). The sign of maturity is that the fruit surface is full yellow of ‘Mituoluo’ and full red of ‘Chuanzhihong’. Sampling was performed in the morning (09:00–10:00 a.m.) under sunny weather conditions. After harvest, fruits with uniform size, color, and free from visible mechanical damage, disease, or pest infection were selected. The samples were randomly divided into five groups and subjected to different temperature treatments (0, 5, 10, 15, and 25 °C). Samples were collected at 0, 1, 3, and 5 days during storage, and an additional sampling point at day 6 after recovery treatment (return to 25 °C) was included. For each sampling point, three biological replicates were collected, and each biological replicate included multiple fruits. For metabolite and enzyme activity analyses, each biological replicate was measured with at least three technical replicates to ensure data reliability. For molecular analyses, tissues were rapidly frozen in liquid nitrogen and stored at −80 °C until further use.

### 2.2 Identification and analysis of characteristic volatile compounds in apricot fruit

Mixed samples of peel and pulp were collected from six fruit each from Mituoluo and Chuanzhihong trees at the green stage, turning color stage, and mature stage for metabolome analysis. All samples were immediately frozen in liquid nitrogen and stored at −80 °C until analysis. Prior to extraction, frozen tissue was ground to a fine powder in liquid nitrogen, and approximately 1 g of the powder was used for extraction. Each sample was combined with 5 ml 20% (*w*/*v*) NaCl and 1 μl methyl isobutyl carbinol (0.1%, *v*/v) was added as the internal standard and allowed to macerate at 25 °C for 4 h. For each developmental stage and low-temperature treatment, three biological replicates were analyzed. Each replicate consisted of a pooled sample of six fruit, and three independent extractions were performed per sample. Volatile compounds were measured using headspace solid phase microextraction (SPME). Following equilibration at 40 °C for 30 min, a DVB/CAR/PDMS (50/30 μm) fiber was exposed to the headspace for 30 min and desorbed in the gas chromatograph (GC) injector for 8 min (Wang et al., 2023). An Agilent 7890B GC instrument with an autosampler system, coupled with an Agilent 5977 A mass spectrometer (MS, Agilent Technologies, Santa Clara, CA, USA), was used to analyze volatile compounds in these samples. The volatiles were separated on an HP-INNOWAX capillary column (60 m × 0.25 mm id, 0.25 μm film thickness) (J&W Scientific, Folsom, CA, USA). The oven conditions were as follows: 50 °C for 1 min, raised to 220 °C at a rate of 3 °C min^−1^, held for 5 min. Helium was the carrier gas (1 ml min^−1^). Mass spectra were recorded in electron impact mode (*m/z* 30–350). Compounds were identified by comparison with the NIST library and retention indices.

Three biological replicates were analyzed per stage and genotype. Relative contents of volatile compounds, including phenylethanol and cymene, were calculated based on GC–MS peak areas. For each sample, the peak area of a given compound was divided by the sum of the peak areas of all detected volatile compounds and expressed as a fraction of the total. Volatile compounds were identified by comparing their mass spectra with those in the NIST/EPA/NIH Mass Spectral Library (NIST 14), and only matches with high similarity scores (>800) were considered reliable (**Table S6**). In addition, compound identification was further supported by comparison of retention times (RT) and calculated Kovats indices (KI) with reference values from the NIST database. The Kovats index was calculated based on a series of n-alkanes under the same chromatographic conditions. Compounds showing close agreement between calculated KI and reference KI values were considered confidently identified.

### 2.3 Transcriptome analysis of apricot fruit during ripening

We sampled 6 fruits at different developmental stages, temperature treatments, and storage periods separately and mixed the tissues of 6 fruits separately for RNA sequencing. Total RNA was extracted from Mituoluo and Chuanzhihong fruit samples at three developmental stages (green stage, turning color stage, and mature stage) and mature fruit subjected to low-temperature treatment (0, 5, 10, 15, or 25 °C) for different periods (0, 1, 3, or 5 d, or 5 d with a 1-d recovery period at 25 °C [6 d]) for transcriptome analysis. Total RNA was extracted from ‘Mituoluo’ and ‘Chuanzhihong’ apricot fruit at three developmental stages (green stage, turning color stage, and mature stage), as well as from mature fruit stored at low-temperature (0, 5, 10, or 15 °C) or room temperature (25 °C). Low-temperature treatments were applied for 0, 1, 3, or 5 d, and an additional recovery treatment consisted of 5 days of low-temperature storage followed by 1 day at 25 °C.) Sequencing libraries were generated using an NEBNext Ultra RNA Library Prep Kit for Illumina (New England Biolabs, MA, USA) according to the manufacturer's manual. The libraries were sequenced on an Illumina HiSeq 2000 instrument (Illumina Inc., San Diego, CA, USA). The raw reads were filtered with fastp software (version 0.19.7) by removing reads containing adapter, poly-N, and low-quality reads (Chen et al., 2018). The filtered reads were aligned to the *Prunus armeniaca* genome (https://www.rosaceae.org/species/prunus_armeniaca/genome_v1.0) using HISAT2 software (Kim et al., 2015). Based on the genome position of mapping reads, StringTie software was applied to assemble transcript (Pertea et al., 2015). The corresponding gene sequences of assembled transcript were annotated in KEGG, GO, NR, Swiss-Prot, TrEMBL, and KOG database with diamond software (Buchfink et al., 2015). Gene expression levels were calculated using the fragments per kilobase of transcript per million mapped reads (FPKM) method by featureCounts software (Liao et al., 2014). EdgeR software was used to conduct differential expression analysis in each pairwise comparison and perform multiple hypothesis testing correction on the probability of the null hypothesis (*P* value) using the Benjamini-Hochberg method to obtain false discovery rate (FDR). The criteria for determine differentially expressed genes (DEGs) were |log_2_ Fold Change | ≥ 1 and a FDR < 0.05 (Robinson et al., 2010). The FPKM values of all the combined DEGs were standardized using the scale function in Rstudio, and then K-means clustering analysis was performed. Heatmap of gene expression levels was plotted using TBtools with the ‘Log Scale’ and ‘Row Scale’ packages (Chen et al., 2020). The clusterProfiler R software package was used for KEGG pathway enrichment analyses of the DEGs (Wu et al., 2021).

### 2.4 Metabolomic analysis of volatile compounds in apricot fruit stored at different temperatures

A total of 50 kg of disease-free, undamaged mature fruit from Mituoluo or Chuanzhihong trees with uniform ripeness were randomly divided into five treatment groups and stored at 90–95% relative humidity. The fruit was stored at 0, 5, 10, 15, 25 °C, and samples were collected at 0, 1, 3, or 5 d, or 5 d with a 1-d recovery at 25 °C [6 d] after cold storage, with six biological replicates per time point per treatment and per genotype. For each sample, 20 g of homogenized fruit tissue was used. For each treatment, three independent biological replicates were analyzed. Metabolomic analysis was conducted as described in Section 2.2.

### 2.5 Measuring LOX activity under different low-temperature storage conditions

Mature fruit from Chuanzhihong and Mituoluo trees was stored at 0, 5, 10, 15, or 25 °C. Samples were collected at 0, 1, 3, or 5 d, or 5 d with a 1-d recovery at 25 °C [6 d] after cold storage before measuring LOX enzyme activity. LOX activity was assayed using a Lipoxygenase Activity Detection Kit (Solarbio, BC0320, Beijing, China) following the manufacturer's protocol. Approximately 0.1 g of tissue was homogenized on ice in 1 ml of LOX extraction buffer, followed by centrifugation at 16,000 ×*g* for 20 min at 4 °C. The supernatant was collected as the crude enzyme extract. The determination and calculation of LOX activity were performed using a LOX activity assay kit (BC0320, Beijing Solarbio Science & Technology, Beijing, China).

### 2.6 Determination of alcohol acyltransferase (AAT) activity under different low-temperature storage conditions

Mature fruit from Chuanzhihong and Mituoluo trees was stored at 0, 5, 10, 15, or 25 °C and sampled at 0, 1, 3, or 5 d, or 5 d with a 1-d recovery at 25 °C [6 d], before measuring AAT activity. For each treatment, six fruit were cut in half, and one half (approximately 3 g) was chopped into small pieces and homogenized in 6 ml of extraction buffer supplied with the Acyltransferase (AAT) Activity Assay Kit (BC2350; Beijing Solarbio Science & Technology, Beijing, China). The homogenate was centrifuged at 15,000 ×*g* for 20 min at 4 °C, and the supernatant was collected to measure enzymatic activity. Distilled water was used as the blank. For each sample, 40 μl of enzyme extract was placed into the well of a microplate and mixed with 10 μl of 98% (*v*/v) 1-octen-3-ol, and 2 μl of 40% (v.v) acetaldehyde. The reaction was immediately measured using a microplate reader at 412 nm, and absorbance values were recorded at 0 s (A₁) and 300 s (A₂). AAT activity was defined as the amount of enzyme that catalyzed a change of 0.0005 in absorbance units per minute per gram of tissue at 37 °C. ΔA_empty = A2_empty − A1_empty; ΔA_measured = A2_measured − A1_measured; ΔA = ΔA_measured − ΔA empty. The activity was calculated using the following formula: AAT (U/g mass) = ΔA ÷ 0.0005 ÷ (V sample ÷ V total sample × W) ÷ T × (V total reverse ÷ 1) = 10,000 × ΔA ÷ W.

### 2.7 Statistical analysis

Data analysis was conducted using the Student's *t*-test and one-way analysis of variance (ANOVA). A difference between the means was considered statistically significant if *p* < 0.05. All data presented in the figures are shown as means ±standard error (SE). All analyses were performed with SPSS statistics software (Version 16.0, SPSS Inc., Chicago, IL, United States).

## 3 Results

### 3.1 Ester compounds are key components of the characteristic volatile aroma of ‘Mituoluo’ fruit

**Fig. 1:**
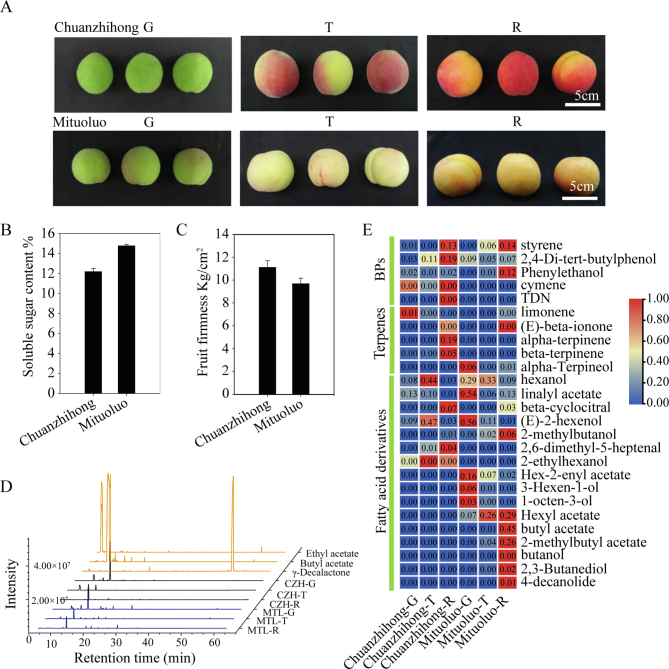
Formation and accumulation of volatile flavor compounds in Chuanzhihong and Mituoluo apricot fruits at the green, turning color stage, and mature stages. (A) Representative photographs of Chuanzhihong and Mituoluo fruits at the green (G), color transition (T), and mature (R) stages. (B) Soluble solids contents of Chuanzhihong and Mituoluo fruits at the mature stage. Values are means ± standard error (SE) from 3 replicates. (C) Firmness of Chuanzhihong and Mituoluo fruits at the mature stage. Values are means ± SE from 3 replicates. (D) Representative gas chromatography–mass spectrometry (GC–MS) chromatograms of volatile compounds from Chuanzhihong and Mituoluo fruits at the green, color transition, and mature stages. (E) Heatmap representation of the contents of benzene derivatives (BPs), terpenoids, and fatty acid-derived volatile compounds detected in Chuanzhihong and Mituoluo fruit at the green (G), color transition (T), and mature (R) stages. (For interpretation of the references to color in this figure legend, the reader is referred to the web version of this article.)

Apricot fruit development can be classified into the green stage, turning color stage, and mature stage based on fruit appearance (Fig. 1A). The soluble solids content of Mituoluo fruit at the mature stage was 14%, whereas that of Chuanzhihong fruit was 12% (Fig. 1B). Mituoluo fruit was softer, with a firmness of 9 kg/cm^2^, compared to 11 kg/cm^2^ for Chuanzhihong fruit (Fig. 1C).

Chuanzhihong and Mituoluo fruits released low levels of volatile compounds at the green stage and the turning color stage, as revealed by GC–MS analysis. However, as they matured, Mituoluo fruit released abundant volatile compounds, with multiple peaks appearing at retention times from 20 min to 40 min (Fig. 1D). We detected 27 volatile compounds in these mature apricots (Fig. 1E**, Table S1**), including benzene derivatives, terpenoids, and fatty acid derivatives. We detected five benzene aromatic compounds in fruit: 2,4-di-tert-butylphenol, phenylethanol, cymene, styrene, and naphthalene (TDN). Among these volatile compounds, we only detected TDN in mature Chuanzhihong fruit, which has a faint aroma. The phenylethanol and cymene contents showed little change during fruit development, with relative contents of 0.1311 and 0.1854, respectively, calculated as the fraction of each peak area over the sum of all volatile compound peak areas. Styrene and 2,4-di-tert-butylphenol levels gradually increased during fruit development, indicating that styrene and 2,4-di-tert-butylphenol are important components of benzene aromatic volatile compounds in apricot fruit.

We detected five terpenoid compounds during the development of Chuanzhihong and Mituoluo fruits: limonene, α-terpineol, β-terpinene, α-terpinene, and (*E*)-β-ionone. The relative contents of these compounds were low, ranging from 0.0006 to 0.047, indicating that terpenoids are not the main contributors to volatile compounds in apricot fruit (**Table S1**).

The most abundant volatile compounds detected during Chuanzhihong and Mituoluo fruit development were 17 fatty acid derivatives (such as butyl acetate, hexyl acetate, linalyl acetate, 2,3-butanediol, 2-methylbutyl acetate, hex-2-enyl acetate, 4-nonanolide, 4-decanolide), as well as alcohols and aldehyde compounds (such as butanol, 2-methylbutanol, 2-ethylhexanol, hexenol, 3-hexen-1-ol, 1-octen-3-ol, β-cyclocitral, and 2,6-dimethyl-5-heptenal). During apricot fruit development, few volatile compounds were released at the green stage and turning color stage. As the fruit matured further, we detected abundant volatile compounds, with the two varieties releasing contrasting types of compounds at different levels. Mature Chuanzhihong and Mituoluo fruits produced styrene, hexanol, 2,4-di-tert-butylphenol, linalyl acetate, β-cyclocitral, (E)-2-hexenol, 2-methylbutanol, 2-methylbutyl acetate, phenylethanol, limonene, and (E)-β-ionone. By contrast, we only detected β-terpinene, α-terpinene, 2,6-dimethyl-5-heptenal, cymene, 2-ethylhexanol, and TDN in mature Chuanzhihong fruit. Similarly, only Mituoluo fruit released butanol, 3-hexen-1-ol, 1-octen-3-ol, α-terpineol, hex-2-enyl acetate, hexyl acetate, butyl acetate, 2,3-butanediol, and 4-decanolide.

The compounds with relatively high abundance in mature Chuanzhihong fruit were styrene, 2,4-di-tert-butylphenol, and α-terpinene, with relative contents of 0.1311, 0.1854, and 0.1897, respectively. By contrast, the compounds with relatively high abundance in mature Mituoluo fruit were butyl acetate, hexyl acetate, 2-methylbutyl acetate, styrene, linalyl acetate, and 4-decanolide, with relative contents of 0.4502, 0.2911, 0.2576, 0.1363, 0.1294, and 0.006, respectively. Olfactory threshold analysis (Van Gemert, 2011) of these volatile compounds showed that 2,4-di-tert-butylphenol and α-terpinene have higher thresholds of 0.1 mg/m^3^ and 0.01 mg/m^3^, respectively. On the contrary, butyl acetate had a lower olfactory threshold of 0.001 mg/m^3^; hexyl acetate had an olfactory threshold of 0.002 mg/m^3^, 2-methylbutyl acetate had an olfactory threshold of 0.0004 mg/m^3^, and 4-decanolide had an olfactory threshold of 0.008 mg/m^3^ (Van Gemert, 2011). Based on these results, the ester compounds hexyl acetate, butyl acetate, 2-methylbutyl acetate, and 4-decanolide are the main volatile compounds in Mituoluo apricot fruit. Therefore, we focused our analysis on the effects of low-temperature treatment on these compounds.

### 3.2 Transcriptome analysis of apricot fruit at the green, color transition, and mature stages and identification of key genes for ester volatile compound formation

**Fig. 2:**
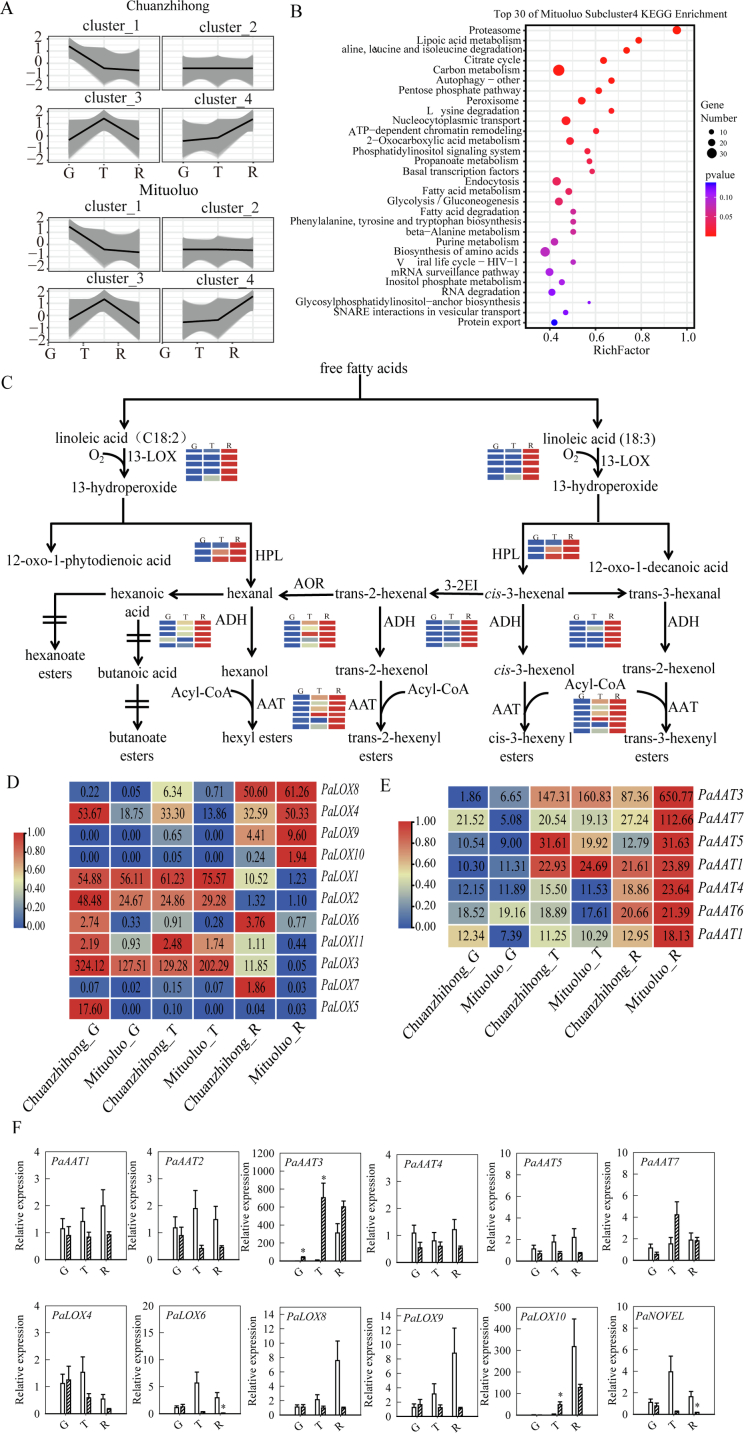
Transcriptome analysis of differentially expressed genes in apricot fruit at the green, color transition, and mature stages. (A) Clustering of differentially expressed genes (DEGs) in Chuanzhihong and Mituoluo fruits at the green (G), transition (T), and mature (R) stages. (B) Top 30 KEGG pathways enriched in DEGs from cluster 4. (C) Summary of the biosynthetic pathway of fatty acid derivatives for ester biosynthesis showing the transcript levels of *LOX*, *HPL*, *ADH*, and *AAT* family genes. (D, E) Heatmap representation of transcript levels for *LOX* (D) and *AAT* (E) family genes in apricot fruit at the green, color transition, and mature stages. (F) RT-qPCR analysis of the candidate gene during fruit development. Asterisks indicate significant differences between the two cultivars at fruit development stages (*p* < 0.05). (For interpretation of the references to color in this figure legend, the reader is referred to the web version of this article.)

We performed transcriptome deep sequencing (RNA-seq) of Chuanzhihong and Mituoluo fruits during maturation, covering the green, color transition, and mature stages. We then identified DEGs across fruit development, defining four modules based on expression patterns (Fig. 2A). Cluster 1 contains 7212 DEGs from Chuanzhihong fruit and 7153 DEGs from Mituoluo fruit; these genes were highly expressed at the green stage, before declining during fruit maturation. Cluster 2 contains 5101 DEGs from Chuanzhihong fruit and 9852 DEGs from Mituoluo fruit, with stable expression levels at the green stage, turning color stage, and mature stage. Cluster 3 consists of 4926 DEGs from Chuanzhihong fruit and 4439 DEGs from Mituoluo fruit; these genes were expressed at low levels at the green and mature stages and at high levels at the turning color stage. Cluster 4 comprises 11,066 DEGs from Chuanzhihong fruit and 6862 DEGs from Mituoluo fruit; these genes were not expressed or were expressed at low levels at the green stage, after which their expression levels gradually increased at the turning color stage, reaching high levels at the mature stage.

Of these four clusters, the expression patterns of DEGs in cluster 4 were consistent with the pattern of volatile compound release in Mituoluo fruit. Among the top 30 KEGG pathways enriched in DEGs from cluster 4 (Fig. 2B), the major metabolism-related pathways included the citrate cycle (tricarboxylic acid [TCA] cycle), carbon metabolism, pentose phosphate pathway, propanoate metabolism, fatty acid metabolism, fatty acid degradation, phenylalanine, tyrosine and tryptophan biosynthesis, beta-alanine metabolism, and inositol phosphate metabolism. Fatty acid metabolism and fatty acid degradation are closely related to volatile compounds in apricot fruit. Linoleic acid and linolenic acid serve as substrates in the biosynthesis of fatty acid derivatives for ester biosynthesis (Fig. 2C), which is catalyzed by LOX to form hydroperoxides. These hydroperoxides are used to synthesize aldehydes and alcohols via reactions catalyzed by HPL and ADH. In turn, aldehydes and alcohols are used to form esters via reactions catalyzed by AATs.

The expression patterns of multiple genes in the *LOX*, *HPL*, *ADH*, and *AAT* gene families were consistent with the release patterns of ester compounds during fruit development (Fig. 2C). LOX-type enzymes mainly act on unsaturated fatty acids in plants, catalyzing oxidation reactions of double bonds to generate hydroperoxides which are core precursors for the subsequent generation of various volatile compounds. AAT catalyzes acetylation reactions between alcohol compounds and acetyl-CoA to generate acetate esters and CoA, serving as a key enzyme in the final step of ester biosynthesis. Therefore, we focused our analysis on *LOX* and *AAT* genes. All genes in the *LOX* family were expressed during fruit development, of which six showed a low-to-high expression pattern from the green to mature stages (Fig. 2D). Similarly, six *AAT* genes showed a low-to-high expression pattern during fruit development (Fig. 2E). Additionally, to further validate the biological hypothesis and explore potential regulatory factors, the expression pattern of the candidate gene was examined by RT-qPCR during fruit development (Fig. 2F). The results showed that expressions of *PaAAT3* and *PaAAT7* was markedly higher in ‘Mituoluo’ than in ‘Chuanzhihong’, with particularly strong induction observed at 25 °C and during the mature stage. This expression pattern was consistent with the RNA-seq data and paralleled the accumulation trends of aroma-related metabolites.

In summary, we identified over 20,000 DEGs at the green, color transition, and mature stages of apricot fruit maturation and grouped them into four clusters. The DEGs in cluster 4 were not expressed or were expressed at low levels at the green stage, expressed at higher levels at the transition stage, and highly expressed at the mature stage; this pattern is consistent with the biosynthesis and accumulation patterns of fruit volatile compounds. Most of the top 30 KEGG pathways enriched for DEGs from cluster 4 were related to metabolism, including fatty acid metabolism and fatty acid degradation. In the biosynthetic pathway of fatty acid derivatives leading to ester biosynthesis, six *LOX* and six *AAT* genes showed a low-to-high expression pattern from the green to mature stages.

### 3.3 Effects of different low-temperature treatments on key flavor compounds in mature Chuanzhihong and Mituoluo fruits during storage

**Fig. 3:**
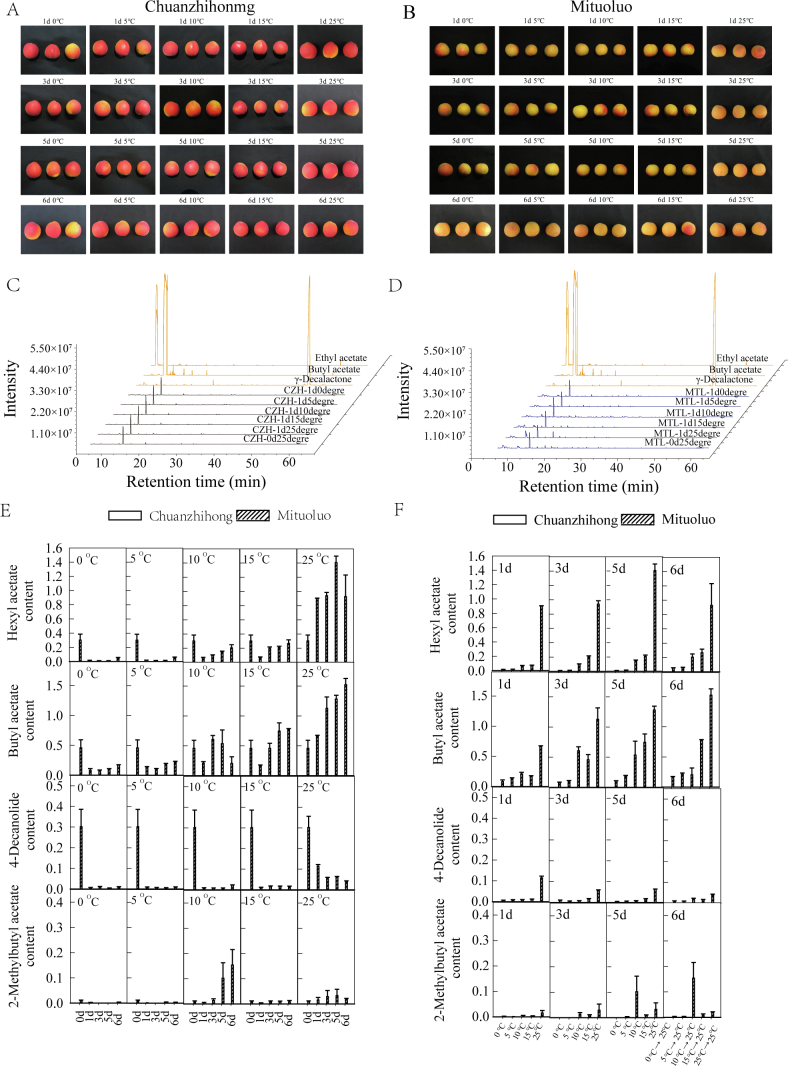
Effects of low-temperature storage on volatile compound release in mature Chuanzhihong and Mituoluo apricot fruits. (A, B) Representative photographs of Chuanzhihong (A) or Mituoluo (B) fruits during storage at 0, 5, 10, 15, or 25 °C for the indicated times. 6 d, 5 days of cold storage, followed by 1 day of recovery at 25 °C. (C, D) Representative GC–MS chromatograms of Chuanzhihong (C) or Mituoluo (D) whole fruit. (E, F) Changes in the relative contents of the major volatile esters hexyl acetate, butyl acetate, 2-methylbutyl acetate, and 4-decanolide under different storage temperatures and durations, plotted as a function of temperature (E) or storage duration (F). Values are means ± SE from three replicates.

The decay rates of mature Chuanzhihong fruit were 39.1% and 50% after five and six days of storage at 25 °C, respectively. For mature Mituoluo fruit, the decay rates were 52.6% and 100% after five and six days of storage at 25 °C, respectively. Based on these observations, we limited the duration of room temperature treatment to a maximum of 7 days for subsequent experiments to avoid excessive fruit decay. We stored mature Chuanzhihong and Mituoluo fruits at different temperatures (0, 5, 10, 15, or 25 °C) for 0 d (as control, CK), 1, 3, or 5 d, alone or with a 1-d recovery at 25 °C after cold treatment (6 d). We analyzed key characteristic flavor compounds in samples at each time point (Fig. 3A, B). The major characteristic volatile compounds of apricot fruit were esters, such as hexyl acetate, butyl acetate, 2-methylbutyl acetate, and 4-decanolide. Therefore, we analyzed these characteristic volatile compounds in mature Chuanzhihong and Mituoluo fruits stored at different temperatures (Fig. 3C, D).

Hexyl acetate is a key volatile compound in apricot fruit. However, we only detected this compound in mature Mituoluo fruit, but not in mature Chuanzhihong fruit (Fig. 3E, F**, Table S2**). When fruit was stored at 25 °C, the amount of hexyl acetate that they released gradually increased with longer storage time, reaching a peak at 5 d with a relative content of 1.4051 and beginning to decline at 6 d. Compared to the pattern observed at 25 °C, the hexyl acetate content in Mituoluo fruit was lower after low-temperature treatment. When stored at 0 or 5 °C, the hexyl acetate content of Mituoluo fruit gradually decreased with longer treatment time, from 0.3016 to 0.0107 and 0.0106, respectively. The hexyl acetate content increased after 5 d of treatment followed by 1 d of recovery at 25 °C but remained lower than that at 25 °C. When fruit was stored at 10 or 15 °C, the amount of hexyl acetate released by Mituoluo fruit gradually increased with longer treatment time. After 5 d of treatment followed by 1 d of recovery at 25 °C, the amount of hexyl acetate released increased but still did not reach the level of the control samples maintained at 25 °C. These results indicate that after storing mature Mituoluo fruit at lower temperatures (0 or 5 °C), followed by recovery at 25 °C, the amount of hexyl acetate released increases but remains lower than that of fruit stored at room temperature. We obtained similar results for Mituoluo fruit stored at 10 or 15 °C, followed by recovery at 25 °C for 1 d (Fig. 3E, F).

Butyl acetate, another key volatile compound in apricots, accumulated to high levels only in mature Mituoluo fruit (Fig. 3E, F**, Table S3**). At 25 °C, the release of butyl acetate continuously increased with longer storage time, with relative content rising from 0.4565 to 1.5224. The butyl acetate content of Mituoluo fruit decreased when the storage temperature was 0 °C relative to that at 25 °C. The butyl acetate content was much lower when fruit was stored at 0 or 5 °C, with relative content dropping from 0.4565 to 0.0806 and 0.0923, respectively. After 5 d of treatment at 0 or 5 °C, followed by 1 d of recovery at 25 °C, the amount of butyl acetate that was released rose but was still lower than that released by fruit stored at 25 °C. When fruit was stored at 10 or 15 °C, the amount of butyl acetate released by Mituoluo fruit was lower than at 25 °C, with the lowest relative contents of 0.2078 and 0.1744, respectively.

Likewise, after 5 d of cold treatment followed by 1 d of recovery at 25 °C, the amount of butyl acetate that was released did not change. Therefore, lower temperature (0 or 5 °C) treatment caused the greatest drop in relative butyl acetate content in Mituoluo fruit. After a 1-d recovery period at 25 °C, the amount of released butyl acetate increased but remained lower than that seen in fruit maintained at room temperature. More butyl acetate was released in mature Mituoluo fruit stored at 10 or 15 °C and its levels remained stable after recovery at 25 °C (Fig. 3**, Table S3**).

Another characteristic volatile component of apricot fruit is 4-decanolide, which we detected only in mature Mituoluo fruit (Fig. 3E, F**, Table S4**). When stored at 25 °C, the relative content of 4-decanolide continuously decreased with longer storage time, from 0.3016 to 0.0391. After low-temperature treatment, the 4-decanolide content of mature Mituoluo fruit was lower than that of fruit maintained at 25 °C. The 4-decanolide content of Mituoluo fruit gradually declined with longer treatment times at 0, 5, and 10 °C. Even though the release of 4-decanolide increased after 5 d of treatment followed by 1d of recovery at 25 °C when compared to the 5-d treatment period alone, its level of release was still lower than that from fruit maintained at room temperature. In fruit stored at 15 °C, the relative content of 4-decanolide in Mituoluo fruit increased slightly over time. After 5 d of treatment followed by 1 d of recovery at 25 °C, the amount of 4-decanolide increased but remained lower than that in fruit kept at 25 °C. These results indicate that the release of 4-decanolide increases after storage at lower temperatures and recovery at 25 °C. However, the total amount of 4-decanolide released is far lower than that of fruit stored at 25 °C (Fig. 3**, Table S4**).

We detected 2-methylbutyl acetate in mature Mituoluo fruit regardless of storage conditions (Fig. 3E, F**, Table S5**). At 25 °C, the release of 2-methylbutyl acetate gradually increased with longer storage time, reaching a maximum value of 0.0324 on day 5. After storage at 0 or 5 °C, the 2-methylbutyl acetate content of Mituoluo fruit was lower than that at 25 °C. No 2-methylbutyl acetate was detected after 3 or 5 d of treatment at 0 °C. In fruit stored at 10 °C, the relative content of 2-methylbutyl acetate was 0.1015 on day 5 and increased to 0.1540 after 1 d of recovery at room temperature. In fruit stored at 15 °C, the relative content of 2-methylbutyl acetate gradually decreased over time, from 0.0108 to 0.0044 on day 1, to 0.0100 on day 3, and to 0.0080 on day 5. After 1 d of recovery at room temperature, the relative content of 2-methylbutyl acetate remained at 0.0082. These results indicate that 10 °C is the optimal temperature for maintaining the 2-methylbutyl acetate content in Mituoluo fruit.

We thus determined that low temperature has a marked effect on the release of volatile compounds in mature fruit. At lower storage temperatures (0 or 5 °C), the release of volatile compounds in Mituoluo fruit was very low or even undetectable after prolonged low-temperature treatment. Even after 5 d of low-temperature treatment followed by 1 d of recovery at 25 °C, the relative contents of volatile compounds increased but were still much lower than those at 25 °C. Based on these results, we conclude that low temperature affects the release of volatile flavor compounds in apricot fruit. Once mature apricot fruit have been stored at 0 to 5 °C, their flavor will degrade even after they are returned to room temperature. When the fruit is placed at 10–15 °C, the release of volatile compounds is higher than that at 0 or 5 °C, and the relative contents of volatile compounds even increased after 1 d of recovery at room temperature.

### 3.4 Effects of low-temperature storage on LOX and AAT transcript levels and LOX and AAT enzyme activities in mature Chuanzhihong and Mituoluo fruits

**Fig. 4:**
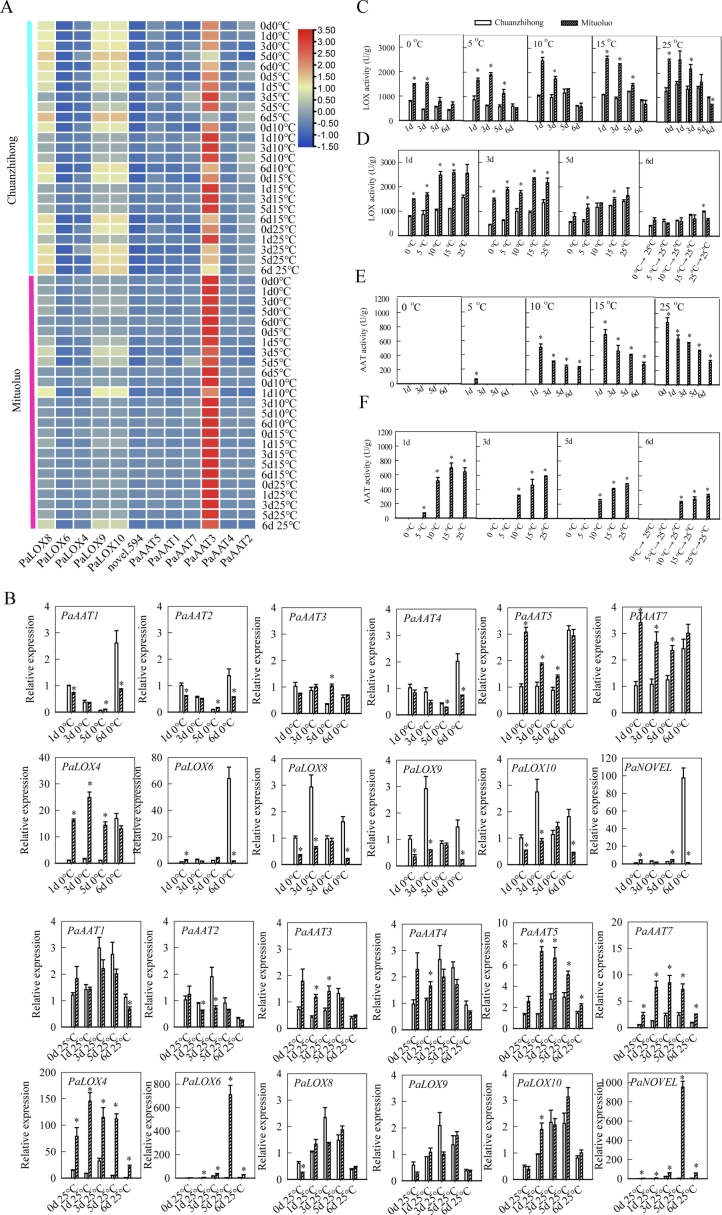
Effects of low-temperature storage on *LOX* and *AAT* transcript levels and LOX and AAT enzymatic activities in mature apricot fruits of ‘Chuanzhihong’ and ‘Mituoluo’. (A) Heatmap showing relative expression levels of LOX and AAT genes in fruits stored at different temperatures and durations. (B)Validation of RNA-seq data by RT-qPCR analysis of aroma-related genes in apricot fruits. (C, E) Changes in LOX (C) and AAT (E) activity as a function of treatment duration at different storage temperatures. (D, F) Changes in LOX (D) and AAT (F) activity as a function of storage temperature at different treatment durations. In B—F, values are means ± SE from 3 replicates. Asterisks indicate significant differences between the two cultivars at the same temperature and time point (*p* < 0.05).

Mituoluo apricot fruit contains flavor compounds such as hexyl acetate, butyl acetate, 4-decanolide, and 2-methylbutyl acetate. Mituoluo apricot fruit contains flavor compounds such as hexyl acetate, butyl acetate, 4-decanolide, and 2-methylbutyl acetate. These flavor compounds are closely associated with lipoxygenase (LOX) enzymes, which play a key role in the biosynthesis of volatile compounds. To further investigate this, we measured the relative expression levels of six LOX genes and six AAT genes in mature apricot fruit under different low-temperature storage conditions. We measured the relative expression levels of six *LOX* genes and six *AAT* genes in mature apricot fruit under different low-temperature storage conditions. Among *LOX* genes, the expression level of *PaLOX8* in Chuanzhihong and Mituoluo fruits increased with longer treatment time at 25 °C, from 52.4 to 55.5 FPKM and from 47.2 to 126.3 FPKM, respectively. By contrast, *PaLOX4* expression levels decreased from 14.8 to 5.9 FPKM in Chuanzhihong fruit and from 31.1 to 19.7 FPKM in Mituoluo fruit. The expression levels of all *LOX* genes were low when mature Chuanzhihong and Mituoluo fruits were stored at 0 or 5 °C. *LOX* gene expression showed a temperature-dependent response, with low levels at 0–5 °C, increased expression at 10–15 °C, and pronounced repression at 25 °C in both cultivars (Fig. 4A). Five *AAT* genes showed decreased expression with longer storage at 25 °C, among which *PaAAT3* was expressed at relatively high levels. This gene was differentially expressed between Chuanzhihong and Mituoluo fruits at 25 °C, with expression values of 83.3 and 405.8 FPKM, respectively. When mature Chuanzhihong and Mituoluo fruits were stored at 0 or 5 °C, *PaAAT3* transcript levels decreased significantly, ranging from 13.9 to 63.65 FPKM in Chuanzhihong fruit and from 57.7 to 260.9 FPKM in Mituoluo fruit.The expression levels of *PaAAT3* in Chuanzhihong and Mituoluo fruits at 10 and 15 °C showed distinct patterns, with Mituoluo exhibiting higher expression than Chuanzhihong. In fruit stored at 25 °C, the expression level of *PaAAT7* in both Chuanzhihong and Mituoluo increased with longer treatment time, from 4.35 to 11.0 FPKM in Chuanzhihong fruit and from 13.7 to 20.4 FPKM in Mituoluo fruit. We detected little difference in *PaAAT7* expression levels between Chuanzhihong and Mituoluo fruits under different low-temperature storage conditions.

To validate the reliability of the RNA-seq data, RT-qPCR analysis was performed on 12 selected genes associated with volatile ester and lipid-derived aroma biosynthesis, including *AAT1–AAT5, AAT7, LOX4, LOX6, LOX8–LOX10,* and *NOVEL* (Fig. 4B). The expression profiles of these genes were examined in two apricot cultivars (‘Chuanzhihong’ and ‘Mituoluo’) under 0 °C and 25 °C storage conditions at four time points (1, 3, 5, and 6 days). Overall, the RT-qPCR results showed expression trends highly consistent with those obtained from RNA-seq, confirming the robustness and reliability of the transcriptomic data. Among the analyzed genes, *AAT3*, *AAT5* and *AAT7*, which are key enzymes involved in ester biosynthesis, exhibited markedly higher expression levels in ‘Mituoluo’ compared with ‘Chuanzhihong’ under both temperature treatments. Similarly, lipid metabolism-related genes *LOX4* and *LOX6* also showed significantly enhanced expression in ‘Mituoluo’, particularly at later storage stages. In addition, the candidate gene *NOVEL* displayed a dramatically elevated expression level in ‘Mituoluo’, especially under 25 °C conditions, suggesting its potential role in regulating aroma compound accumulation during fruit ripening. In contrast, most genes showed relatively lower or stable expression levels in ‘Chuanzhihong’, indicating cultivar-dependent differences in aroma-related metabolic activity. Taken together, these results further support that ‘Mituoluo’ has a stronger capacity for aroma biosynthesis, which is consistent with the metabolomic observations and reinforces the biological conclusions of this study.

We plotted LOX enzyme activity in mature Chuanzhihong and Mituoluo fruits as a function of the temperature treatment (Fig. 4C**)**. LOX activity reached a peak in mature Chuanzhihong and Mituoluo fruits stored at 25 °C for 1 d, with values of 1560.5 U/g and 2546.7 U/g, respectively. LOX activity gradually declined with longer storage periods, reaching the lowest value at 6 d for Chuanzhihong fruit (990.43 U/g) and Mituoluo fruit (664.57 U/g). When mature Chuanzhihong and Mituoluo fruits were stored at different low temperatures (0, 5, 10, or 15 °C), LOX activity decreased with decreasing storage temperature. Among temperature treatments, the highest LOX activities in fruit stored at 0 or 5 °C were 1883.7 U/g and 862.4 U/g, respectively, which were much lower than those in fruit stored at 10 or 15 °C, where the highest activities were 2572.7 and 1097.4 U/g, respectively. LOX activity decreased with longer treatment periods: the lowest activity was detected at 6 d (5 d of low-temperature treatment followed by 1 d of recovery at 25 °C). Therefore, LOX activity in mature Chuanzhihong and Mituoluo fruits did not increase significantly after 1 d of recovery at room temperature following low-temperature storage.

We then plotted LOX activity in mature Chuanzhihong and Mituoluo fruits as a function of treatment time (Fig. 4D). LOX activity increased with higher temperature and was lower in Chuanzhihong fruit than in Mituoluo fruit. The LOX activity of Chuanzhihong fruit was 783.7, 862.4, 1038.7, and 1097.4 U/g under treatment at 0, 5, 10, or 15 °C for 1 day, respectively, compared to 1560.5 U/g for fruit maintained at 25 °C for 1 d. For Mituoluo fruit, LOX activity was 1476.9, 1665.9, 2478.8, and 2572.7 U/g following a 1-day treatment at 0, 5, 10, or 15 °C, and 2546.7 U/g for fruit maintained at 25 °C for the same period, with no significant differences in LOX activity among the 10, 15, and 25 °C treatments. We observed similar patterns in Chuanzhihong and Mituoluo fruits after storage for 3 or 5 d. When mature fruit was stored at 0, 5, 10, or 15 °C for 5 d followed by 1 d of recovery at 25 °C (6 d), LOX activity in Chuanzhihong fruit increased with higher temperature, whereas LOX activity showed little change in Mituoluo fruit. These results indicate that both temperature and treatment duration affect LOX activity in Chuanzhihong and Mituoluo fruits, with activity detected even in fruit stored at 0 and 5 °C. There were no significant differences in LOX activity among 10, 15, and 25 °C treatments, which was more similar to that at 25 °C. The LOX activity of mature Chuanzhihong and Mituoluo fruits did not increase significantly after 1 d of recovery at room temperature following low-temperature storage.

As with LOX activity, we plotted AAT enzyme activity in mature Chuanzhihong and Mituoluo fruits as a function of the temperature treatment (Fig. 4E). There was no detectable AAT activity in Chuanzhihong fruit under any cold treatment. For Mituoluo fruit, AAT activity gradually decreased with longer treatment at 25 °C, from 872.7 U/g at 0 d, 640.0 U/g at 1 d, 581.5 U/g at 3 d, 484.4 U/g at 5 d, to 318.7 U/g at 6 d. When mature Mituoluo fruit was stored at 0 °C, no AAT activity was detected regardless of cold treatment duration, even after 5 d of treatment followed by 1 d of recovery at 25 °C. When Mituoluo fruit was stored at 5 °C, we detected a low level of AAT activity (63.0 U/g) at 1 d, with no activity detected under other treatment conditions. When mature Mituoluo fruit was stored at 10 or 15 °C, AAT enzyme activity gradually decreased with longer treatment, as observed under 25 °C treatment. These results indicate that low temperature (0–5 °C) leads to undetectable AAT activity even after 1 d of recovery at 25 °C. When the treatment temperatures rose to 10 and 15 °C, AAT activity in apricot fruit followed the same release pattern as at 25 °C.

Finally, we plotted AAT enzyme activity in mature Chuanzhihong and Mituoluo fruits as a function of temperature for each treatment duration (Fig. 4F). No AAT activity was detected in Chuanzhihong fruit under any treatment. When Mituoluo fruit was subjected to different temperature treatments for 1 d, AAT activity increased with higher temperature, reaching the highest value of 696.0 U/g at 15 °C. After 3 d of low-temperature treatment of Mituoluo fruit, AAT activity increased from 0 (0–5 °C) to 581.5 U/g at 25 °C. We obtained similar patterns of AAT activity after 5 d of low-temperature treatment, with the highest value of 484.4 U/g at 25 °C. When mature Mituoluo fruit as stored at 0, 5, 10, or 15 °C for 5 d followed by 1 d of recovery at 25 °C (6 d), AAT activity was undetectable in the 0 and 5 °C samples, and lower than that of fruit that had remained at 25 °C in the other groups, ranging from 228.5 to 318.7 U/g.

A comprehensive analysis of the above results revealed that both temperature and treatment duration have significant effects on enzymatic activities in Chuanzhihong and Mituoluo fruits. These activities were the lowest, or even undetectable, at storage temperatures of 0 °C and 5 °C. However, the enzymatic activities exhibited similar patterns and values in fruit stored at 10, 15, or 25 °C. The enzyme activities of mature Chuanzhihong and Mituoluo fruits did not increase significantly after 1 d of recovery at room temperature following a period of low-temperature treatment.

### 3.5 Principal component analysis and correlation analysis of volatile esters, enzymatic activities, and structural gene expression in apricot fruit

**Fig. 5:**
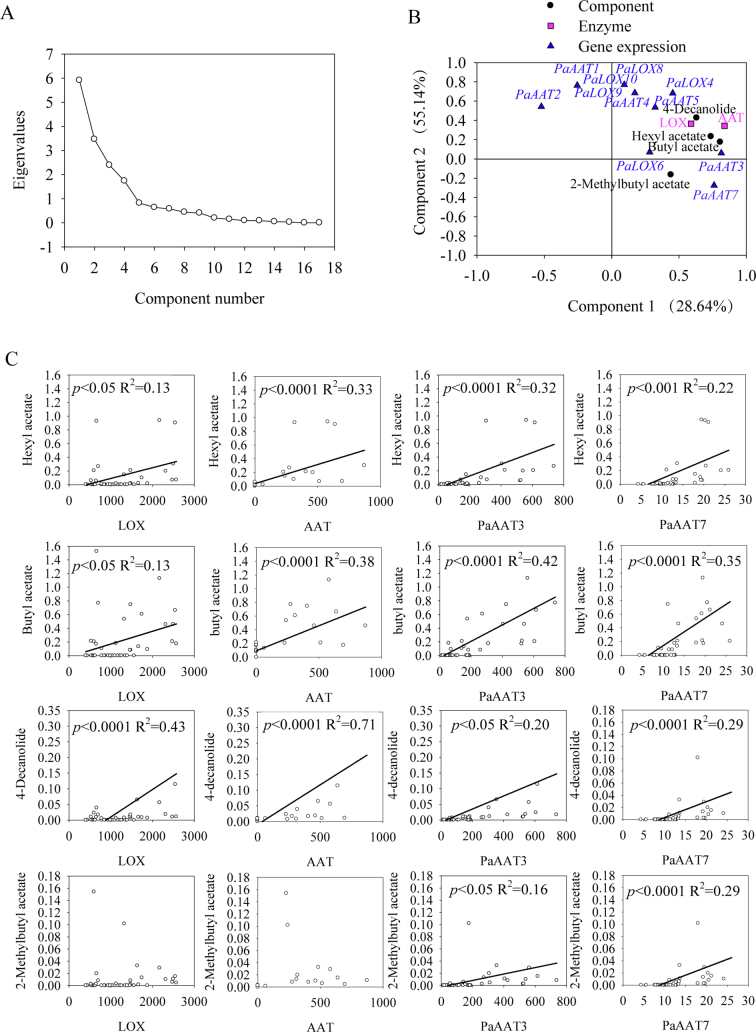
Principal component analysis and correlation analysis of the content of volatile ester compounds, enzymatic activities, and structural gene expression in apricot fruit. **(A)** Scree plot showing eigenvalues of principal components derived from the principal component analysis (PCA). **(B)** PCA plot of the first two principal components (PC1 and PC2) after varimax rotation, integrating the content of ester volatile compounds, LOX and AAT enzymatic activities, and *PaLOX* and *PaAAT* expression levels. **(C)** Pearson's correlation analysis between the contents of four representative ester compounds (hexyl acetate, butyl acetate, 4-decanolide, and 2-methylbutyl acetate) and enzymatic activities (LOX and AAT) or *PaAAT* expression. Solid lines represent linear regression fits, with corresponding *P* values and *R*^2^ indicated.

We conducted principal component analysis (PCA) to examine the correlations among the concentrations of major ester volatiles, LOX and AAT enzymatic activities, and the transcript levels of *PaLOX* and *PaAAT* structural genes involved in aroma formation in apricot fruit. We also generated a scree plot to assess how many significant components to focus on (Fig. 5A). The first two principal components (PCs) explained most of the total variance and were therefore selected for analysis. The varimax rotation was applied to enhance the interpretability of the principal components by maximizing the variance of the loadings and reducing overlap among variables. After varimax rotation, PC1 mainly represented ester-related variation, whereas PC2 was primarily associated with LOX-related variables (Fig. 5B). Typical ester compounds, including hexyl acetate, butyl acetate, 4-decanolide, and 2-methylbutyl acetate, showed high positive loadings along PC1. AAT activity was also strongly aligned with PC1, together with the transcript abundance of *PaAAT7* and *PaAAT3*, indicating that variation in ester accumulation was closely associated with AAT catalytic activity and the expression levels of specific *AAT* genes. By contrast, PC2 was mainly characterized by LOX-associated variables, with positive loading for LOX activity and the expression of *PaLOX8*, *PaLOX9*, and *PaLOX10* along PC2. Notably, *PaAAT1*, *PaAAT2*, and *PaAAT4* were also distributed in the upper region of PC2, suggesting coordinated variation between LOX-derived precursor formation and downstream esterification-related genes. Together, PC1 and PC2 revealed a clear clustering pattern among ester volatiles, enzymatic activities, and *LOX* and *AAT* expression, reflecting the integrated variation of metabolic and expression features underlying aroma formation in apricot fruit.

Based on the results of the PCA, we selected four representative ester compounds (hexyl acetate, butyl acetate, 4-decanolide, and 2-methylbutyl acetate) for correlation analysis with enzymatic activities (LOX and AAT) and structural gene expression (Fig. 5C). The content of hexyl acetate showed a significant positive correlation with AAT activity (*r* = 0.575, *P* < 0.001), whereas its correlation with LOX activity was not significant. In addition, the content of hexyl acetate was significantly and positively correlated with *PaAAT3* (*r* = 0.565, *P* < 0.001) and *PaAAT7* expression levels (*r* = 0.47, *P* < 0.001), suggesting a close association with variation in the transcript levels of these *AAT* genes. The content of butyl acetate exhibited a similar correlation pattern, showing a strong positive correlation with AAT activity (*r* = 0.62, *P* < 0.001) and significant associations with *PaAAT3* (*r* = 0.65, *P* < 0.001) and *PaAAT7* expression levels (*r* = 0.60, *P* < 0.001). These results suggest shared regulatory features between ester formation at the C4 and C6 positions. Among the analyzed esters, 4-decanolide content displayed the strongest positive correlation with AAT activity (*r* = 0.845, *P* < 0.001). 4-Decanolide content was also significantly correlated with LOX activity (*r* = 0.65, *P* < 0.001) and with *PaAAT3* (*r* = 0.45, *P* < 0.05) and *PaAAT7* expression levels (*r* = 0.54, *P* < 0.0001), indicating that both the supply of LOX-mediated precursors and AAT-related reactions were associated with its accumulation. By contrast, 2-methylbutyl acetate content showed no significant correlation with LOX or AAT activity but was positively correlated with *PaAAT3* (*r* = 0.39, *P* = 0.005) and *PaAAT7* expression levels (*r* = 0.53, *P* < 0.001), suggesting a stronger dependence on transcript variation of specific *AAT* genes.

These results demonstrate that the contents of characteristic volatile compounds in apricot fruit, including hexyl acetate, butyl acetate, 4-decanolide, and ethyl 2-methylbutanoate, are closely correlated with the transcript levels of genes such as *PaAAT7* and *PaAAT3* as well as AAT enzymatic activity. Collectively, these findings indicate that the expression levels of *PaAAT7*, *PaAAT3*, and homologous genes, together with AAT activity, are key determinants governing the emission of the characteristic volatiles in apricot fruit.

## 4 Discussion

During apricot fruit development, few volatile compounds are released at the green stage or turning color stage, but a rich variety of volatile substances can be detected as the fruit ripens. The aroma of apricot fruit that we perceive is a mixture of multiple volatile compounds. Different apricot cultivars produce distinct fruit volatile substances. Extensive research has been conducted to detect volatile compounds in apricot fruit worldwide. For example, a study of five French apricot cultivars (‘Iranien’, ‘Orangered’, ‘Goldrich’, ‘Hargrand’, and ‘Rouge du Roussillon’) and the hybrid progeny A4025 revealed that their primary fruit volatile compounds are ethyl acetate, hexyl acetate, limonene, cyclocitral, decalactone, 6-methyl-5-hepten-2-one, linalool, β-ionone, menthone, and (*E*)-hexen-2-al, to name a few (Guillot et al., 2006). Twenty-six volatile compounds were identified in the French cultivar ‘Bergeron’. Among these, γ-decalactone, δ-undecalactone, γ-nonalactone, α-decalactone, (E)-β-damascenone, and (*R*/*S*)-linalool were the major volatile substances. In addition, â-ionone, (Z)-1,5-octadien-3-one, ç-decalactone, (E,Z)-2,6-nonadienal, linalool, and acetaldehyde serve as key characteristic aroma compounds of ‘Bergeron’ apricot fruit (Greger & Schieberle, 2007). SPME–GC–MS technology was employed to detect volatile compounds in several Turkish apricot cultivars (‘Hasanbey’, ‘Hacihaliloglu’, ‘Kabaasi’, ‘Soganci’, ‘Hacikiz’, ‘Cataloglu’, ‘Cologlu’, and ‘Aprikoz’). Ethanol, hexanal, hexyl acetate, (Z)-3-hexenyl acetate, (E)-2-hexenyl acetate, 1-hexanol, (Z)-3-hexenol, and (E)-2-hexen-1-ol were identified in these Turkish cultivars at various levels (Gokbulut & Karabulut, 2012). The major volatile compounds in fruit from the Australian apricot cultivar ‘Castlebrite’ are ethyl acetate, hexanal, linalool, and trans-β-ionone (Defilippi et al., 2009).

In China, the Xinjiang region is an important source and cultivation center for apricots, with abundant wild and cultivated germplasm resources (Zhou et al., 2021). Analysis of volatile compounds in five major apricot cultivars in Xinjiang (‘Kuerle Tuoyong’, ‘Akeyaleke’, ‘Kezijianali’, ‘Suogejianali’, and ‘Sulian No. 2’) at full ripeness revealed that γ-decalactone, δ-decalactone, δ-dodecalactone, hexyl acetate, cis-3-hexenyl acetate, β-damascenone, and dihydro-β-ionone are the primary characteristic aroma components of their fruit (Lu et al., 2016). In addition, propyl acetate, 3-methyl-1-butanol acetate, (Z)-3-hexen-1-ol acetate, d-limonene, b-linalool, hexanal, hexyl acetate, butyl acetate, b-myrcene, ethyl butanoate, and b-cis-ocimene were identified as the major aroma components in 14 Xinjiang apricot cultivars by high-performance (HP)–SPME–GC–MS (Feng et al., 2015). In the current study, we measured volatile compounds in the fruit of the highly aromatic apricot cultivar Mituoluo. We detected 27 volatile substances, among which 2,4-di-tert-butylphenol, styrene, butyl acetate, hex-2-enyl acetate, hexyl acetate, and 4-decanolide were the major aroma components in these fruits.

During apricot fruit development, a climacteric response occurs, which is characterized by a sudden increase in respiratory rate accompanied by the massive release of ethylene. This climacteric response promotes rapid fruit softening, peel coloration, and flavor formation. Since apricot fruit is prone to decay after ripening, low-temperature storage and preservation are commonly used in production. However, this storage method often leads to the loss of volatile flavor compounds and reduces fruit quality after ripening, leading to consumer dissatisfaction (Defilippi et al., 2009). Defilippi et al. determined that mature fruit of the apricot cultivar Castlebrite contain high levels of ethyl acetate, hexenal, and (E)-2-hexenal. Following storage at 0 °C for 15 d or 30 d, the contents of these compounds decreased significantly. In particular, ethyl acetate became undetectable after 30 d of storage at 0 °C (Defilippi et al., 2009). Another study found that when fruits stored at near-freezing temperatures were transferred to a shelf environment at 20 °C for 4 days, the activities of HPL, ADH and AAT could recover to 70%–80% of the control levels (Liu et al., 2019). However, this is inconsistent with our research, which the recovery effect was negligible when the fruits were placed at 25 °C for only 1 day.

In the current study, mature apricot fruit was subjected to storage at 0–10 °C. There was minimal release of volatile compounds such as hexyl acetate, butyl acetate, 2-methylbutyl acetate, and 4-decanolide in Mituoluo fruit under these conditions. After allowing the fruit to recover at room temperature (25 °C) for 1 day, the release of these volatile substances increased but remained significantly lower than that of fruit stored continuously at 25 °C. Under low-temperature treatment at 15 °C, the release of key volatile compounds (hexyl acetate, butyl acetate, 2-methylbutyl acetate, and 4-decanolide) in Mituoluo fruit increased with longer storage duration but remained lower than the level released at 25 °C. Clearly, low temperatures affect the release of volatile flavor compounds in apricot fruit. When mature apricot fruit is stored at 0–10 °C, even if it is subsequently placed at room temperature, its flavor is irreversibly lost. By contrast, under low-temperature treatment at 10–15 °C, the release of volatile compounds from apricot fruit was significantly higher than that from fruit stored at 0–10 °C. Notably, the release of volatile substances even increased after transferring the fruit to room temperature following storage at 10–15 °C. Therefore, low temperatures in the range of 10–15 °C help maintain the aromatic quality of apricot fruit.

Volatile compounds play a crucial role in determining fruit quality. However, the molecular mechanisms underlying aroma regulation, particularly under cold storage conditions, remain insufficiently understood. Increasing evidence suggests that low temperature can markedly influence volatile production by modulating key metabolic pathways at both metabolic and transcriptional levels. For example, Peng et al. (2024) investigated low temperature–induced aroma loss in the highly aromatic ‘Golden Empress’ Hami melon and reported a dramatic decrease in fruity ester content from 782.8 μg/kg at room temperature to 53.7 μg/kg after storage at 3 °C, indicating strong inhibition of ester biosynthesis. Transcriptomic analysis further revealed significant changes in 52 genes associated with volatile biosynthesis, with key genes such as alcohol dehydrogenase (*ADH*), long-chain acyl-CoA synthetase (*ACSL*), and branched-chain amino acid aminotransferase (*ilvE*) significantly downregulated under low temperature (Peng et al., 2024). Similarly, in banana, cold storage suppresses the production of aroma-related volatiles, particularly esters, by regulating key genes involved in amino acid and fatty acid metabolism, including those in the lipoxygenase (LOX) pathway (Zhu et al., 2018). In peach (*Prunus persica* L.), low temperature not only leads to chilling injury but also significantly reduces volatile accumulation. Transcriptomic analyses have demonstrated that genes associated with the LOX pathway are highly responsive to temperature changes and play essential roles in aroma formation (Zhang et al., 2011). Consistent patterns have also been reported in strawberry (*Fragaria* × *ananassa*), where low-temperature storage (4–8 °C) leads to widespread transcriptional reprogramming. Most transcription factor families are downregulated, whereas NAC and WRKY families are upregulated, accompanied by significant downregulation of alcohol acyltransferase (AAT), a key enzyme in ester biosynthesis, thereby affecting volatile release (Baldwin et al., 2023). In apricot, transcriptomic studies have suggested that multiple transcription factors and structural genes are involved in aroma biosynthesis (Zeng et al., 2025). The accumulation of aroma compounds in the cultivar ‘Modesto’ has been shown to be closely associated with the expression of AAT, pyruvate decarboxylase (PDC), LOX, and ADH genes, with AAT expression markedly increasing during fruit ripening (González-Agüero et al., 2009). Moreover, during low-temperature storage of ‘Xiaobai’ apricot fruit, ester and alcohol contents decline significantly, and the expression levels of LOX and AAT are positively correlated with ester accumulation, indicating that esters are key determinants of postharvest fruit quality (Pei et al., 2023). In the present study, low-temperature treatment of ‘Chuanzhihong’ and ‘Mituoluo’ fruits resulted in significant repression of both the expression and enzymatic activities of LOX and AAT, which are critical for ester biosynthesis. Notably, at 0 °C and 5 °C, LOX and AAT activities reached minimal or even undetectable levels. These results demonstrate a clear link between transcriptional regulation, enzyme activity, and volatile accumulation, providing mechanistic insight into how low temperature inhibits aroma formation in apricot fruit.

In conclusion, our study demonstrates that low-temperature storage affects volatile composition in apricot fruit by key aroma-related enzymes. As storage temperature decreases, both the diversity and abundance of volatile compounds decline markedly, with some compounds becoming undetectable at 0–5 °C. Notably, these changes are not fully reversible after transfer to room temperature, indicating a persistent inhibitory effect of low temperature on aroma formation. Importantly, low temperature suppresses ester biosynthesis primarily through coordinated downregulation of key genes and enzymes involved in the lipoxygenase (LOX) pathway and alcohol acyltransferase (AAT)-mediated reactions. When the temperature lowers to 0–5 °C, some volatile compounds even become undetectable. More types and higher levels of volatile compounds are detected at storage temperatures above 10 °C than at 0–5 °C. The activities of related enzymes cannot be restored to control levels after cold exposure below 10 °C, even after being returned to room temperature (25 °C). Together, these findings establish a mechanistic link between transcriptional regulation, enzyme activity, and volatile metabolite profiles in apricot fruit under cold storage. Our findings shed light on the mechanism by which low-temperature storage affects aroma formation in mature apricot fruit, providing a methodological and practical foundation for the storage management of mature apricot fruit and offering a theoretical basis for improving the postharvest quality of apricot fruit in the market.

## CRediT authorship contribution statement

**Hua Wang:** Writing – original draft, Validation, Methodology, Formal analysis, Data curation. **Pei Sun:** Validation, Methodology, Formal analysis, Data curation. **Yuan Yang:** Validation, Methodology. **Wenjian Yu:** Validation, Methodology. **Yanhui Kang:** Validation, Methodology. **Maofu Li:** Validation, Methodology. **Shuting Zhou:** Validation, Methodology. **Xiangyi Sun:** Validation, Methodology. **Min Jin:** Validation, Methodology. **Wanmei Jin:** Writing – review & editing, Writing – original draft, Supervision, Funding acquisition, Conceptualization. **Haoyuan Sun:** Writing – review & editing, Supervision, Funding acquisition, Conceptualization. **Yuzhu Wang:** Writing – review & editing, Supervision, Funding acquisition, Conceptualization.

## Fundings

This work was supported by the Key 10.13039/100006190Research and Development Program Project of Xinjiang Autonomous Region (2024B02019–3) and the BAAFS Scientific Research Project (KJCX20230602).

## Declaration of competing interest

The authors declare the following financial interests/personal relationships which may be considered as potential competing interests: Wanmei Jin reports financial support was provided by Beijing Academy of Agriculture and Forestry Sciences. Wanmei Jin, Haoyuan Sun, and Yuzhu Wang has patent Method for preserving the flavor of stored apricots pending to 202,410,164,456.5. If there are other authors, they declare that they have no known competing financial interests or personal relationships that could have appeared to influence the work reported in this paper.

## Data Availability

Data will be made available on request.
