# Supplementary material for: Profiling major volatile components in apricot fruit sheds light on the molecular mechanisms underlying low-temperature-mediated volatile release
Source: Food Chem (Oxf). 2026 May 9;12:100409. doi: 10.1016/j.fochms.2026.100409 (PMC13195593; doi:10.1016/j.fochms.2026.100409)
Supplement: Supplementary file 1 — Supplementary material [file mmc1.docx]

Supplementary Table 1: Formation and Accumulation of Aroma and Flavor Compounds during the Green, Coloring, and Maturation Stages of Chuanzhihong and Mituoluo Fruits

|  | Compound Name | Chuanzhihong | Mituoluo | Chuanzhihong | Mituoluo | Chuanzhihong | Mituoluo |
| --- | --- | --- | --- | --- | --- | --- | --- |
|  |  | Green stage | Green stage | Turning stage | Turning stage | Ripe stage | Ripe stage |
| Benzenoid-phenylpropanoids | styrene | 0.0094±0.0011 | 0.0036±0.0003 | 0.0047±0.0013 | 0.0649±0.0065 | 0.1311±0.0667 | 0.1363±0.0330 |
|  | 2,4-Di-tert-butylphenol | 0.0261±0.0046 | 0.0851±0.0059 | 0.1089±0.0444 | 0.0525±0.0030 | 0.1854±0.0772 | 0.0686±0.0136 |
|  | Phenylethanol | 0.0205±0.0008 | 0 | 0.0139±0.0056 | 0.0148±0.0070 | 0.0199±0.0089 | 0.1206±0.0884 |
|  | cymene | 0.0019±0.0019 | 0 | 0.0006±0.0006 | 0 | 0.0024±0.0020 | 0 |
|  | TDN | 0 | 0 | 0 | 0 | 0.0032±0.0014 | 0 |
| Terpenes | limonene | 0.0111±0.0028 | 0 | 0.0017±0.0017 | 0 | 0.0006±0.0001 | 0.0025±0.0008 |
|  | (E)-beta-ionone | 0 | 0 | 0 | 0 | 0.0023±0.0003 | 0.0032±0.0019 |
|  | alpha-terpinene | 0 | 0 | 0 | 0 | 0.1897±0.0940 | 0 |
|  | beta-terpinene | 0.0025±0.0024 | 0 | 0.0047±0.0034 | 0 | 0.0539±0.0531 | 0 |
|  | alpha-Terpineol | 0 | 0.0629±0.0034 | 0 | 0.0047±0.0014 | 0 | 0.0133±0.0040 |
| Fatty acid derivatives | hexanol | 0.0824±0.0184 | 0.2854±0.1284 | 0.4444±0.1546 | 0.3297±0.1222 | 0.0267±0.0146 | 0.0910±0.0043 |
|  | linalyl acetate | 0.1340±0.0284 | 0.5408±0.0191 | 0.1000±0.0255 | 0.0573±0.0120 | 0.0095±0.0095 | 0.1294±0.0308 |
|  | beta-cyclocitral | 0.0030±0.0003 | 0.0014±0.0007 | 0.0042±0.0009 | 0.0026±0.0013 | 0.0685±0.0376 | 0.0331±0.0082 |
|  | (E)-2-hexenol | 0.0885±0.0662 | 0.5598±0.2601 | 0.4657±0.1416 | 0.1107±0.0176 | 0.0342±0.0262 | 0.0097±0.0075 |
|  | 2-methylbutanol | 0 | 0.0035±0.0035 | 0 | 0.0161±0.0080 | 0.0050±0.0041 | 0.0565±0.0411 |
|  | 2,6-dimethyl-5-heptenal | 0.0012±0.0006 | 0 | 0.0127±0.0058 | 0 | 0.0435±0.0303 | 0 |
|  | 2-ethylhexanol | 0.0015±0.0002 | 0 | 0.0034±0.0006 | 0 | 0.0026±0.0021 | 0 |
|  | Hex-2-enyl acetate | 0 | 0.1629±0.0094 | 0 | 0.0737±0.0368 | 0 | 0.0191±0.0125 |
|  | 3-Hexen-1-ol | 0 | 0.0639±0.0033 | 0 | 0.0065±0.0010 | 0 | 0.0026±0.0018 |
|  | 1-octen-3-ol | 0 | 0.0279±0.0030 | 0 | 0.0040±0.0016 | 0 | 0.0009±0.0006 |
|  | Hexyl acetate | 0 | 0.0702±0.0044 | 0 | 0.2644±0.1296 | 0 | 0.2911±0.1302 |
|  | butyl acetate | 0 | 0 | 0 | 0.0059±0.0006 | 0 | 0.4502±0.0914 |
|  | 2-methylbutyl acetate | 0 | 0 | 0 | 0.0352±0.0209 | 0 | 0.2576±0.1475 |
|  | butanol | 0 | 0 | 0 | 0 | 0 | 0.0022±0.0011 |
|  | 2,3-Butanediol | 0 | 0 | 0 | 0.0015±0.0015 | 0 | 0.0233±0.0095 |
|  | 4-decanolide | 0 | 0 | 0 | 0 | 0 | 0.0060±0.0030 |

Supplementary Table 2: Effect of Low-Temperature Treatment on Hexyl Acetate in Maturity Stage Fruits of Chuanzhihong and Mituoluo.

| Treatment | 0d | | 1d | | 3d | | 5d | | Recovery at Room Temperature for 1 Day | |
| --- | --- | --- | --- | --- | --- | --- | --- | --- | --- | --- |
|  | Chuanzhihong | Mituoluo | Chuanzhihong | Mituoluo | Chuanzhihong | Mituoluo | Chuanzhihong | Mituoluo | Chuanzhihong | Mituoluo |
| 0°C | 0 | 0.3016±0.0844 | 0 | 0.0171±0.0006 | 0 | 0.0122±0.0005 | 0 | 0.0107±0.0015 | 0 | 0.0545±0.0026 |
| 5°C | 0 | 0.3016±0.0844 | 0 | 0.0199±0.0012 | 0 | 0.0151±0.0015 | 0 | 0.0160±0.0020 | 0 | 0.0590±0.0056 |
| 10°C | 0 | 0.3016±0.0844 | 0 | 0.0664±0.0053 | 0 | 0.0998±0.0060 | 0 | 0.1441±0.0137 | 0 | 0.2041±0.0448 |
| 15°C | 0 | 0.3016±0.0844 | 0 | 0.0689±0.0080 | 0 | 0.1982±0.0195 | 0 | 0.2081±0.0191 | 0 | 0.2652±0.0524 |
| 25°C | 0 | 0.3016±0.0844 | 0 | 0.9011±0.00408 | 0 | 0.9384±0.0485 | 0 | 1.4051±0.0925 | 0 | 0.9271±0.3050 |

Note: Compound Name, Hexyl acetate; CAS No., 142-92-7; Formula, C8H16O2; Component RT, 17.991.

Supplementary Table 3: Effect of Low-Temperature Treatment on butyl acetate in Maturity Stage Fruits of Chuanzhihong and Mituoluo.

| Treatment | 0d | | 1d | | 3d | | 5d | | Recovery at Room Temperature for 1 Day | |
| --- | --- | --- | --- | --- | --- | --- | --- | --- | --- | --- |
|  | Chuanzhihong | Mituoluo | Chuanzhihong | Mituoluo | Chuanzhihong | Mituoluo | Chuanzhihong | Mituoluo | Chuanzhihong | Mituoluo |
| 0°C | 0 | 0.4565±0.1348 | 0 | 0.0806±0.0300 | 0 | 0.0802±0.0060 | 0 | 0.1047±0.0061 | 0 | 0.1706±0.0046 |
| 5°C | 0 | 0.4565±0.1348 | 0 | 0.1315±0.0128 | 0 | 0.0923±0.0205 | 0 | 0.1801±0.0178 | 0 | 0.2132±0.0207 |
| 10°C | 0 | 0.4565±0.1348 | 0 | 0.2078±0.0278 | 0 | 0.6066±0.0658 | 0 | 0.5337±0.2321 | 0 | 0.2044±0.1127 |
| 15°C | 0 | 0.4565±0.1348 | 0 | 0.1744±0.0049 | 0 | 0.4566±0.0851 | 0 | 0.7444±0.1392 | 0 | 0.7684±0.0205 |
| 25°C | 0 | 0.4565±0.1348 | 0 | 0.6616±0.0146 | 0 | 1.1278±0.1900 | 0 | 1.2808±0.0653 | 0 | 1.5224±0.1051 |

Note: Compound Name, butyl acetate; CAS No., 123-86-4 ; Formula, C6H12O2; Component RT, 10.440.

Supplementary Table 4: Effect of Low-Temperature Treatment on 4-decanolide in Maturity Stage Fruits of Chuanzhihong and Mituoluo.

| Treatment | 0d | | 1d | | 3d | | 5d | | Recovery at Room Temperature for 1 Day | |
| --- | --- | --- | --- | --- | --- | --- | --- | --- | --- | --- |
|  | Chuanzhihong | Mituoluo | Chuanzhihong | Mituoluo | Chuanzhihong | Mituoluo | Chuanzhihong | Mituoluo | Chuanzhihong | Mituoluo |
| 0°C | 0 | 0.3016±0.0844 | 0 | 0.0072±0.0004 | 0 | 0.0107±0.0015 | 0 | 0.0049±0.0003 | 0 | 0.0093±0.0020 |
| 5°C | 0 | 0.3016±0.0844 | 0 | 0.0095±0.0003 | 0 | 0.0068±0.0006 | 0 | 0.0060±0.0008 | 0 | 0.0097±0.0009 |
| 10°C | 0 | 0.3016±0.0844 | 0 | 0.0101±0.0002 | 0 | 0.0079±0.0010 | 0 | 0.0073±0.0016 | 0 | 0.0231±0.0009 |
| 15°C | 0 | 0.3016±0.0844 | 0 | 0.0108±0.0020 | 0 | 0.0181±0.0010 | 0 | 0.0164±0.0020 | 0 | 0.0167±0.0013 |
| 25°C | 0 | 0.3016±0.0544 | 0 | 0.1140±0.0086 | 0 | 0.0557±0.0049 | 0 | 0.0646±0.0001 | 0 | 0.0391±0.0044 |

Note: Compound Name, 4-decanolide; CAS No., 706-14-9; Formula, C10H18O2; Component RT, 50.961.

Supplementary Table 5: Effect of Low-Temperature Treatment on 2-methylbutyl acetate in Maturity Stage Fruits of Chuanzhihong and Mituoluo.

| Treatment | 0d | | 1d | | 3d | | 5d | | Recovery at Room Temperature for 1 Day | |
| --- | --- | --- | --- | --- | --- | --- | --- | --- | --- | --- |
|  | Chuanzhihong | Mituoluo | Chuanzhihong | Mituoluo | Chuanzhihong | Mituoluo | Chuanzhihong | Mituoluo | Chuanzhihong | Mituoluo |
| 0°C | 0 | 0.0108±0.0011 | 0 | 0.0022±0.0004 | 0 | 0.0000±0.0000 | 0 | 0.0000±0.0000 | 0 | 0.0024±0.0015 |
| 5°C | 0 | 0.0108±0.0011 | 0 | 0.0014±0.0000 | 0 | 0.0000±0.0000 | 0 | 0.0030±0.0014 | 0 | 0.0025±0.0010 |
| 10°C | 0 | 0.0108±0.0011 | 0 | 0.0057±0.0002 | 0 | 0.0129±0.0072 | 0 | 0.1015±0.0614 | 0 | 0.1540±0.0615 |
| 15°C | 0 | 0.0108±0.0011 | 0 | 0.0044±0.0001 | 0 | 0.0100±0.0019 | 0 | 0.0080±0.0029 | 0 | 0.0082±0.0054 |
| 25°C | 0 | 0.0108±0.0011 | 0 | 0.0149±0.0103 | 0 | 0.0289±0.0244 | 0 | 0.0324±0.0258 | 0 | 0.0196±0.0023 |

Note: Compound Name, styrene; CAS No., 100-42-5; Formula, C8H8; Component RT,17.361.

Supplementary Table 6: Identification and characterization of major volatile compounds detected by GC–MS in apricot fruits under different temperature treatments.

| Peak NO. | Name | Compound Class | Molecular Formula | Library Match Score | Average Retention Time (RT,min) | Calculated KI | Reference KI(NIST) | RT Variation | Average RT |
| --- | --- | --- | --- | --- | --- | --- | --- | --- | --- |
| 1 | 1-Hydroxy-1-methylcyclopentane | Fatty alcohols | C6H12O | 672 | 4.70 |  | 796.00 | 0.03 | 4.70 |
| 2 | Hexanal | Fatty aldehydes | C6H12O | 906 | 4.84 | 801.12 | 802.00 | 0.05 | 4.84 |
| 3 | n-Butyl acetate | Carboxylic Acid Esters | C6H12O2 | 901 | 5.07 | 811.34 | 812.00 | 0.06 | 5.07 |
| 4 | 2-Hexenal | Fatty aldehydes | C6H10O | 948 | 5.99 | 854.58 | 851.00 | 0.09 | 5.99 |
| 5 | Benzaldehyde | Fatty aldehydes | C7H6O | 942 | 8.54 | 968.17 | 962.00 | 0.00 | 8.54 |
| 6 | (4E)-4-Hexenyl acetate | Carboxylic Acid Esters | C8H14O2 | 841 | 9.47 | 1007.53 | 1006.00 | 0.04 | 9.47 |
| 7 | Hexyl acetate | Carboxylic Acid Esters | C8H16O2 | 896 | 9.62 | 1014.58 | 1011.00 | 0.07 | 9.62 |
| 8 | trans-2-Hexenyl acetate | Carboxylic Acid Esters | C8H14O2 | 779 | 9.68 | 1018.54 | 1016.00 | 0.04 | 9.68 |
| 9 | 4,5-Dimethylnonane | aliphatic hydrocarbons | C11H24 | 747 | 10.52 | 1054.21 | 1056.00 | 0.06 | 10.52 |
| 10 | γ-Decanolactone | Carboxylic Acid Esters | C10H18O2 | 937 | 19.04 | 1475.10 | 1470.00 | 0.07 | 19.04 |
| 11 | 5-Methyltetradecane | aliphatic hydrocarbons | C15H32 | 744 | 19.13 | 1481.72 | 1454.00 | 0.03 | 19.13 |
| 12 | 2-Methyltetradecane | aliphatic hydrocarbons | C15H32 | 767 | 19.20 | 1485.59 | 1463.00 | 0.01 | 19.20 |
| 13 | δ-Amylvalerolactone | Carboxylic Acid Esters | C10H18O2 | 861 | 19.56 | 1503.46 | 1504.00 | 0.08 | 19.56 |
| 14 | 2,4-Di-tert-butylphenol | Phenylpropanoids | C14H22O | 912 | 19.66 | 1511.10 | 1519.00 | 0.01 | 19.66 |
| 15 | 1,3-Pentanediol, 2,2,4-trimethyl-, diisobutyrate | Carboxylic Acid Esters | C16H30O4 | 849 | 20.99 | 1589.78 | 1605.00 | 0.02 | 20.99 |
| 16 | γ-Dodecalactone | Carboxylic Acid Esters | C12H22O2 | 835 | 22.61 | 1688.53 | 1678.00 | 0.03 | 22.61 |
| 17 | 3-Methylhexadecane | aliphatic hydrocarbons | C17H36 | 736 | 22.72 | 1695.93 | 1673.00 | 0.01 | 22.72 |
| 18 | 2-Methylheptadecane | aliphatic hydrocarbons | C18H38 | 740 | 23.41 | 1741.20 | 1765.00 | 0.01 | 23.41 |
| 19 | Hexadecanoic acid, ethyl ester | Carboxylic Acid Esters | C18H36O2 | 873 | 27.10 | 1993.65 | 1993.00 | 0.03 | 27.10 |
| 20 | Heneicosane | aliphatic hydrocarbons | C21H44 | Standard | 28.51 | 2098.50 | 2100.00 | 0.02 | 28.51 |
| 21 | Phytol | Fatty alcohols | C20H40O | 917 | 28.71 | 2114.83 | 2114.00 | 0.06 | 28.71 |
| 22 | Linoleic acid ethyl ester | Carboxylic Acid Esters | C20H36O2 | 905 | 29.31 | 2161.67 | 2162.00 | 0.01 | 29.31 |
| 23 | Linolenic acid, ethyl ester | Carboxylic Acid Esters | C20H34O2 | 710 | 29.39 | 2167.92 | 2171.00 | 0.01 | 29.39 |
| 24 | Tricosane | aliphatic hydrocarbons | C23H48 | Standard | 31.01 | 2298.77 | 2300.00 | 0.01 | 31.01 |
| 25 | Tetracosane | aliphatic hydrocarbons | C24H50 | Standard | 32.18 | 2397.61 | 2400.00 | 0.01 | 32.18 |
| 26 | 1-Docosanal | Fatty aldehydes | C22H44O | 888 | 32.57 | 2432.95 | 2430.00 | 0.01 | 32.57 |
| 27 | Pentacosane | aliphatic hydrocarbons | C25H52 | Standard | 33.30 | 2498.49 | 2500.00 | 0.01 | 33.30 |
| 28 | Hexacosane | aliphatic hydrocarbons | C26H54 | Standard | 34.12 | 2597.75 | 2600.00 | 0.02 | 34.12 |
| 29 | Tetracosanal | Fatty aldehydes | C24H48O | 870 | 34.36 | 2638.21 | 2632.00 | 0.01 | 34.36 |
| 30 | Heptacosane | aliphatic hydrocarbons | C27H56 | Standard | 34.70 | 2698.42 | 2700.00 | 0.01 | 34.70 |
| 31 | Octacosane | aliphatic hydrocarbons | C28H58 | Standard | 35.26 | 2797.01 | 2800.00 | 0.01 | 35.26 |
| 32 | Squalene | aliphatic hydrocarbons | C30H50 | 912 | 35.37 | 2815.37 | 2832.00 | 0.07 | 35.37 |
| 33 | Hexacosanal | Fatty aldehydes | C26H52O | 836 | 35.53 | 2840.17 | 2833.00 | 0.01 | 35.53 |
| 34 | Nonacosane | aliphatic hydrocarbons | C29H60 | Standard | 35.87 | 2898.02 | 2900.00 | 0.01 | 35.87 |
| 35 | 1-Hexacosanol | Fatty alcohols | C26H54O | 838 | 35.92 | 2905.73 | 2906.00 | 0.01 | 35.92 |
| 36 | Triacontane | aliphatic hydrocarbons | C30H62 | Standard | 36.53 | 2997.74 | 3000.00 | 0.01 | 36.53 |
| 37 | Octacosanal | Fatty aldehydes | C28H56O | 802 | 36.89 | 3045.22 | 3039.00 | 0.06 | 36.89 |
| 38 | Hentriacontane | aliphatic hydrocarbons | C31H64 | Standard | 37.29 | 3096.33 | 3100.00 | 0.01 | 37.29 |
| 39 | (±)-α-Tocopherol | Phenylpropanoids | C29H50O2 | 870 | 37.72 | 3146.29 | 3149.00 | 0.01 | 37.72 |
| 40 | 22-Ketocholesterol | ketosteroid | C27H44O2 | 781 | 38.92 | 3267.23 | 3255.00 | 0.04 | 38.92 |
| 41 | Chondrillasterol | sterol | C29H48O | 708 | 39.18 | 3292.73 | 3295.00 | 0.06 | 39.18 |
| 42 | γ-Sitosterol | sterol | C29H50O | 935 | 39.91 | 3355.62 | 3351.00 | 0.02 | 39.91 |
| 43 | Stigmasta-5,24(28)-dien-3-ol, (3β,24Z)- | sterol | C29H48O | 843 | 40.11 | 3372.66 | 3343.00 | 0.03 | 40.11 |
| 44 | Cycloartenol acetate | sterol | C32H52O2 | 753 | 40.38 | 3395.66 | 3389.00 | 0.02 | 40.38 |
| 45 | Cycloartenol | sterol | C30H50O | 869 | 40.84 | 3435.34 | 3466.00 | 0.10 | 40.84 |
| 46 | 24-Methylenecycloartanol | sterol | C31H52O | 750 | 41.47 | 3492.03 | 3108.00 | 0.06 | 41.47 |
| 47 | 9,19-Cyclolanostan-3-ol, 24-methylene-, acetate, (3β)- | sterol | C33H54O2 | 680 | 41.60 | 3502.27 | 3365.00 | 0.03 | 41.60 |
